# Study protocol for a randomized trial examining a peer grief support approach for people grieving drug overdose deaths

**DOI:** 10.1186/s13722-026-00652-8

**Published:** 2026-02-11

**Authors:** Karen Chan Osilla, Katherine E. Watkins, Tanya Lord, Glen Lord, Franklin Cook, Katherine Nameth, Jane P. Kim, Wendy Hawkins, Alison Athey

**Affiliations:** 1 Department of Psychiatry and Behavioral Sciences, Stanford University School of Medicine, 401 Quarry Road, Palo Alto, CA 94305 USA; 2 RAND, 1776 Main Street, PO Box 2136, Santa Monica, CA 90407-2138 USA; 3 Peer Support Community Partners, 3 Sanborn Drive, Nashua, NH 03063 USA; 4 RAND, 1200 S Hayes St, Arlington, VA 22202 USA

**Keywords:** Drug overdose, Death, Peer support, Grief, Family therapy

## Abstract

**Background:**

People bereaved by drug overdose experience a “special grief” that includes guilt, shame, and blame for their loved one’s death, which often compounds suffering and interferes with help-seeking. This population is vulnerable to poor health outcomes including prolonged grief disorder, risky substance use, and suicidal thoughts and behaviors. Peer grief support may reduce risks in people bereaved by overdose by buffering grievers from the negative effects of increased stigma and isolation. Peer Support Community Partners has developed the RIVER peer grief support model for bereavement. The model has been implemented successfully in the community and for drug overdose bereavement, but has not yet been rigorously tested.

**Methods:**

The current study is a two-phased project that aims to test whether the RIVER peer grief support approach improves health outcomes in those grieving drug overdose deaths. In the first phase, we will prepare for and conduct a pilot test of the RIVER peer grief support approach. We will also assess an innovative mechanism for engaging grievers through Medical Examiners Offices (MEOs). In the second phase, we will compare enhanced usual care (EUC) to EUC plus RIVER peer grief support (RIVER) in a randomized controlled trial.

**Conclusions:**

The study aims to build the science of overdose bereavement and provide increased support for a vulnerable population of grievers.

**Trial registration:**

https://clinicaltrials.gov/study/NCT06854757 (02/25/2025).

## Introduction

The United States (US) is in an overdose crisis. An estimated 110,000 Americans died by drug overdose in 2023 [1], putting the national total since 2000 at more than 1.1 million overdose deaths [2]. Overdose death rates have accelerated as the crisis wears on and as more deadly drugs such as fentanyl make their way into the drug supply [3]. The overdose crisis has had profound, negative effects that extend beyond the tragedy of the deaths themselves. For example, the overdose crisis has increased the burden on health, foster care, and criminal-legal systems [4] and spurred on syndemics [5] of suicide [6], infectious disease [7], and violence [8, 9]. Research has focused on the broad effects of the overdose crisis on society but has neglected the impact on people who are left behind by drug overdose deaths and the potential role that overdose bereavement may play in fueling risky substance use among the bereaved [10].

Recent findings highlight it is common for people to lose someone to overdose. About 42% of people in the US (i.e., more than 125 million people) personally know someone who died by overdose [11]. More than 32% of adults report that they have lost a loved one to an opioid overdose [12]. Children have also been heavily impacted by overdose loss. In 2019, more than 1.4 million US children lost a family member to overdose, including 317,000 who lost a parent [13]. To put these data into perspective, the prevalence of exposure to overdose deaths is estimated to be more than three times higher than the prevalence of exposure to suicide [14] and is on par with rates of bereavement by COVID-19 deaths [15]. Without intervention, the ripple effects of overdose loss will persist, affecting millions of individuals and communities.

People who are bereaved by overdose are vulnerable to poor mental health outcomes. They experience high rates of prolonged grief disorder (PGD) [16, 17], a type of grief that lasts longer and has more severe effects than other types of grief [18]. Rates of PGD are higher among those bereaved by overdose compared to those who have lost someone to suicide, homicide, and other unintentional injuries [19]. Overdose bereavement is also associated with the development of posttraumatic stress disorder, depression, anxiety, physical pain [20], as well as suicidal thoughts and behaviors [17, 21–23].

People who experience grief, particularly prolonged grief, are at increased risk for developing their own substance use problems [24–26], perhaps because they struggle to emotionally process and accept the loss [27] or because they use substances to cope. Substance use may fuel substance-related morbidity and mortality among the bereaved by leading some to initiate substance misuse and others to experience increases in the severity of their substance use disorders. Although no research has evaluated the association between overdose bereavement and a subsequent overdose among the bereaved, a study of Norwegian parents who lost children to overdose revealed these grievers were at more than double the risk for external injury deaths (i.e., accidents, suicides, or homicides) [28]. Studies focused on bereaved PWUD show an increased risk for their own experiences of non-fatal or fatal overdose [27, 29]. Overdose bereavement may perpetuate the overdose crisis by increasing risk for overdose morbidity and mortality in people left behind.

The traumatic nature of loss and stigma associated with overdose may make healing from grief more difficult [16, 30]. The Dual Process Model of grief suggests that healing can occur when grievers balance both processing the loss and restoring their lives after loss [21, 30]. People bereaved by overdose may struggle to emotionally process and accept the loss due to intense, negative emotions, including shame and guilt [10, 16, 31–33]. Some may also struggle to accept the loss if there is ambiguity surrounding the circumstances of the death or confusion about whether the overdose was suicidal or homicidal in nature [21, 34]. Research comparing overdose bereavement to other traumatic losses shows that guilt and shame are significantly more severe among people bereaved by overdose than among people bereaved in other ways (e.g., a cancer-related death) [10, 16, 32, 35]. Stigma may be especially common and harmful to people with personal and family histories of drug use [32, 36]. The experience of stigma and intense emotions interfere with meaning making and healing after loss [21] and may contribute to mental health conditions such as depression [17, 31]. Addressing the intense negative emotions that inhibit acceptance of the loss and the experiences of stigmatization may help people bereaved by overdose heal.

People bereaved by overdose are in critical need of resources as they often lack support for restoring their lives after loss [37]. Informal social support from friends and family appears to be a critical component of healthy grieving by promoting the restoration of the lives of bereaved people [37]. Unfortunately, perceived stigma interferes with healing by promoting social withdrawal and negatively impacting the relationships of people bereaved by overdose [32, 38–40]. People bereaved by overdose, especially bereaved PWUD, also face barriers to engaging with professional supports [27]. While most people bereaved by overdose report that they would benefit from professional help, only one-third report they have received it [41]. In the absence of informal and professional support, it can be difficult for those bereaved by overdose deaths to effectively cope with the loss.

Peer grief support interventions may be protective against PGD, substance use, and mental health outcomes. Peer interventions involve “giving and receiving help founded on key principles of respect, shared responsibility, and mutual agreement” [42]. A review found that peer support was associated with reductions in risk for PGD and with improved well-being in people bereaved by sudden or violent deaths [43, 44]. Importantly, people who receive peer grief interventions experience reductions in substance use compared to grievers who do not receive peer support [45–47]. Qualitative studies of this population suggest that peer interventions may serve as an antidote to the trauma and stigma of overdose bereavement by promoting connectedness, acceptance of the loss, and adaptive coping [39, 48]. Research also demonstrates that peer-facilitated interventions can improve outcomes for the facilitators themselves evidenced by greater posttraumatic growth, increased social connectedness and reduced adverse grief outcomes in those facilitating a peer grief intervention [49, 50]. However, the evidence supporting peer grief support interventions for overdose bereavement is scarce. To our knowledge, no peer reviewed research has evaluated the effectiveness of peer interventions for preventing PGD and substance misuse in people who are bereaved by overdose.

**Fig. 1:**
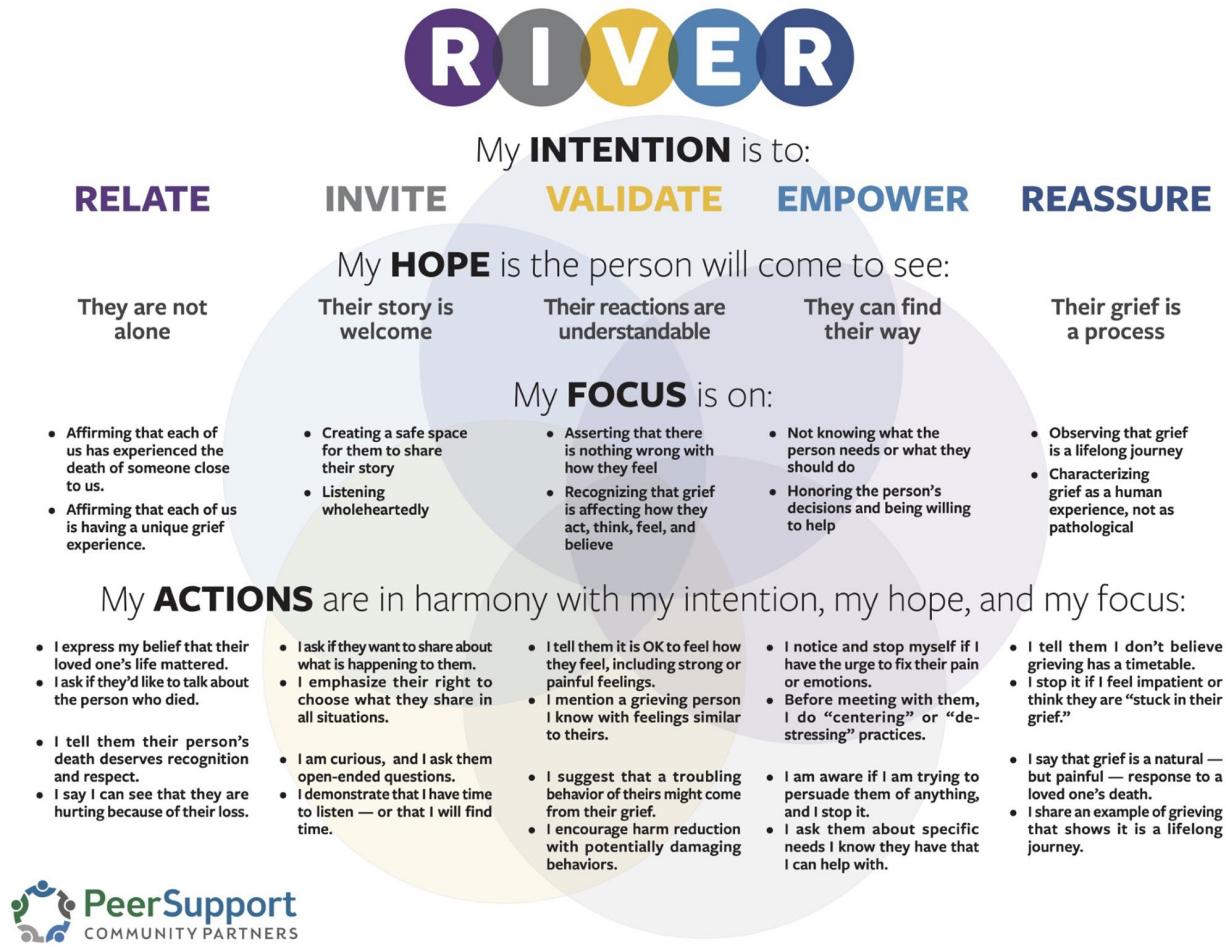
RIVER peer grief support model

To address this gap, researchers from academic institutions have collaborated with a peer grief support organization [i.e., Peer Support Community Partners (PSCP)] in the current study to advance the science of overdose bereavement. PSCP has developed and implemented a peer grief support model called RIVER (relate, invite, validate, empower, reassure) for people bereaved by overdose. The RIVER model (Fig. 1) was developed by people who experienced traumatic bereavement, including bereavement by overdose, after decades of learning from and delivering peer grief support. The RIVER model distills this learning, evidence-based peer support practices, and trauma-informed approaches. Rather than prescribing specific intervention activities, the RIVER model of peer grief support guides peer grief allies, or supporters in *how* to engage with people bereaved by overdose. Peer grief allies are trained to use their lived experience to *relate* to people who are bereaved by overdose, *invite* them to make meaning of the loss by sharing their grief story and demonstrating that their story is welcome, *validate* their grief to show that their reactions are understandable, *empower* grievers to engage in restorative coping so that they can restore their lives after loss, and *reassure* them grief is a lifelong journey and process. While the RIVER peer grief support model was developed without specific attention to the Dual Process Modelof coping with grief, RIVER closely aligns with the model because RIVER promotes balancing both loss- and restoration- related bereavement needs. This study will be the first to test the impact of the RIVER peer grief model on grief, substance use, and mental health outcomes of people bereaved by overdose.

To address evidence that most grievers lack access to grief support, this study established partnerships with Medical Examiners’ Offices (MEOs). MEOs are legally mandated to conduct death investigations following every overdose death and to make death notifications to the legal next-of-kin to those who die by overdose. As part of the death investigation and notification process, MEOs routinely interact with the next-of-kin to overdose decedents. Indeed, MEOs may be the only organizations that systematically compile the contact information of all next-of-kin to overdose decedents within their jurisdictions. Partnering with MEOs may provide a scalable path to proactively engage people bereaved by overdose before they develop poor grief-related outcomes. This study will be the first to evaluate the feasibility, acceptability, and appropriateness of proactively engaging people bereaved by overdose via MEOs.

## Study aims

The current study has three aims. In aim one, we will adapt training materials for the RIVER peer grief support model to fit the needs of the communities surrounding our partnering MEOs, develop a fidelity measure, and create workflows for engaging grievers and connecting them with peer grief allies. In aim two, we will conduct a pilot test to evaluate the acceptability and feasibility of engaging grievers via MEOs, refine the fidelity measure, and evaluate if peer grief support using the RIVER model was satisfactory to grievers. In aim three, we will conduct a randomized controlled trial (RCT) to evaluate the effectiveness of the RIVER peer grief approach plus enhanced usual care (RIVER + EUC) to prevent PGD and substance misuse in people bereaved by overdose, over and above any effects of EUC. Participants will be recruited from MEOs and will complete baseline, 3, 6, and 12-month surveys on (a) proximal outcomes (i.e., connectedness, acceptance, and coping) and (b) long-term outcomes (i.e., PGD and substance misuse). Post-RCT qualitative interviews of study participants and mixed methods assessments with peer grief allies will supplement data collection and allow us to understand the characteristics of the RIVER peer grief approach that drive effectiveness and its impact on peer grief allies.

## Methods

### Overview of study procedures

First, we will supplement the already-developed RIVER peer grief approach with materials needed to rigorously test its effectiveness (Aim 1). We will engage three advisory boards (family, research, and medical examiners) to (1) develop recruitment materials and procedures for recruiting grievers who interact with MEOs (2), adapt RIVER training materials to local MEO regions, and (3) develop fidelity measures. Next, we will conduct a pilot test of the RIVER peer grief support model with at least twenty people who are bereaved by a drug overdose death and recruited through MEOs. We will determine the feasibility and acceptability of griever engagement procedures, as well as grievers’ satisfaction with RIVER peer grief support (Aim 2).

In our third aim, we will use an RCT to test the effects of RIVER plus EUC compared to EUC alone for people who are bereaved by a drug overdose death and recruited from MEOs (Aim 3). People bereaved by a drug overdose death will be randomized to receive RIVER plus EUC or the EUC alone condition. RCT participants (*N* = 320 at baseline; 208 at 12-month follow-up) will be assessed on their experience of proximal and distal grief, substance use, and mental health outcomes at baseline, and during 3-, 6-, and 12-month-follow-ups. We will also conduct qualitative interviews with a subset of participants at the end of the study to contextualize the mechanisms in which the RIVER model affected them. Finally, we will collect qualitative and quantitative data from peer grief allies at the beginning and end of the RCT to explore how delivering peer grief support affects their well-being.

### Advisory boards

We will have three advisory boards that guide our research. The Family Advisory Board will consist of six family members who are bereaved by overdose, many of whom are leaders in community organizations that offer peer grief support. The goals of the FAB will be to give voice to the experiences of people who are bereaved by overdose and to provide strategic guidance on all aspects of the research. The Research Advisory Board will consist of nine members who have expertise within the fields of addiction and overdose bereavement. Their role will be to provide strategic guidance on the study design, appropriateness of the outcomes, analyses, and the interpretation of study results. Finally, the Medical Examiner Advisory Board will consist of seven members and their role will be to provide direction on data exchange and engagement workflows, and enhancement of scalability and sustainability planning of peer grief support in MEOs. We will meet quarterly with each advisory board throughout the study period.

### RIVER peer grief model

Grief work using the RIVER peer grief support model is dynamic and varies based on the individual’s specific circumstances, such as their relationship to the decedent (e.g., whether the relationship was strained) and the circumstances around the death (e.g., whether the death was anticipated). These factors can substantially impact grief and substance use outcomes in the bereaved [21]. At minimum, a RIVER peer grief ally will meet with a griever for at least three 45- to 75-minute virtual sessions. Meetings range in frequency from weekly to monthly (often less over time), and it is common for the meetings to continue for at least a year. These ranges are variable, depending on the wishes and agreement of the griever and the peer grief ally. Pilot data on the number and cadence of peer grief support sessions will be used to determine the minimum and maximum number of peer grief support sessions used in the RCT. In the pilot and RCT, peer grief allies will also provide grievers with the same materials that are part of the EUC, which include a psychoeducational booklet about grief [51] and a local mental health and grief support resource list.

#### Peer grief ally training and supervision

PSCP has a process for onboarding peer grief allies that assesses their readiness to deliver peer grief support given their own bereavement, their willingness to engage in ongoing supervision, their completion of at least 16 h of training, and the results of background checks. Background checks include identity verification and review of state and federal criminal records in accordance with institutional requirements. These checks identify active legal restrictions or unresolved charges that would prohibit participant-facing work. Past justice involvement is not automatically exclusionary; eligibility decisions consider relevance, recency, and potential risk to participant safety. The 16-hour training is broken into two parts. The first half of training focuses on using the RIVER peer grief support approach. Peers are trained in this process model through a combination of didactic information, discussion, and role plays. Peers are trained to identify clinical problems (e.g., posttraumatic stress disorder) and risks (e.g., dangerous substance use, suicidal crises) and taught how to connect people to clinical and emergency services as needed. The second part of the training addresses ways that delivering the RIVER peer grief support model could affect peers’ own well-being and grief process, both negatively through re-traumatization and positively through posttraumatic growth. Peers are informed about well-being resources available through PSCP and are encouraged to use these if they experience re-traumatization or other negative impacts of peer grief work. This portion of the training also includes discussion of regional demographic characteristics, paired with guidance on using a cultural humility approach when supporting participants. Peer allies are encouraged to ask open-ended questions and use reflective listening to understand what is important to each participant, rather than making assumptions based on regional or demographic information. RIVER supervisors meet individually with peers each week to coach them in their delivery of the RIVER peer grief support model and review documentation. As part of this study, supervisors will review and discuss audio recordings of sessions. Supervisors will also explore how grief support delivery impacts the peers’ well-being (e.g., identifying re-traumatization, risky substance use, etc.). Peers are advised to engage with a licensed clinician at PSCP if they experience re-traumatization and to attend monthly group reflective supervision in which they reflect with other peer grief allies on how their work affects their own grief journeys.

#### Enhanced usual care (EUC) for grief support

The EUC condition involves proactive outreach to supply grievers with existing, published psychoeducational booklet about grief and a list of grief support resources in their community in addition to what MEOs provide to grievers. We will provide participants a psychoeducational booklet about grief developed by an organization called *What’s Your Grief* [51]. This 12- page booklet was developed for a lay audience of people bereaved by any sort of death (i.e., not specifically a drug-related death) and has been used by PSCP in their general and overdose specific bereavement work [51]. We will distribute booklets in either English or Spanish, depending on the preference of the griever. We will also provide participants with a list of local grief, mental health, and substance use treatment resources.

### Setting

We will primarily conduct our study in regions that have the highest prevalence of overdose bereavement (i.e., New England and the central Southeast [11]). We will also conduct the study in the Pacific region, where the prevalence of overdose bereavement is lower compared to other US regions, to account for the possibility that grievers experience stigmatization, isolation, and a dearth of grief resources in communities where overdose bereavement is less common. We will partner with MEOs in the state of Connecticut; Jefferson County, AL; and San Diego County, CA. Despite differences in the prevalence of overdose bereavement in these communities, the number of overdose deaths in each region is high. Partnering with MEOs in these locations allows us to bolster bereavement support services in communities deeply impacted by drug overdose deaths.

### Aim 1 procedures

We will take a process-oriented [52] approach to optimizing the RIVER peer grief support model that PSCP has already created, while maximizing both internal and external validity as we prepare for rigorously evaluating the RIVER model’s effectiveness. First, we will refine RIVER training materials. We will work with local MEOs to customize training materials as informed by the local context (e.g., encouraging cultural humility, sharing local trends in next-of-kin demographics, describing how drug use has affected their local community, how to address grievers who use substances themselves). Second, we will develop measures of fidelity and adherence to the RIVER peer grief support model. Like motivational interviewing [53], RIVER is a process model that describes the style in which an intervention is given. Thus, we will develop a RIVER fidelity assessment and coding manual similar to the Motivational Interviewing Treatment Integrity (MITI) measure [54] whereby coders rate peer grief facilitator speech on each of the RIVER domains (relate, invite, validate, empower, reassure) using a 5-point Likert scale. Finally, we will work with our MEO partners to develop recruitment workflows and establish data sharing plans to support proactive outreach to people bereaved by overdose by our research data collection staff.

### Aim 2 procedures

We will work with our three partnering MEOs to recruit 20 people bereaved by overdose for a pilot study of the RIVER peer grief support model (see Fig. 1). We will recruit one bereaved adult per decedent to participate in study assessments but, in keeping with PSCP’s inclusive practice, we will deliver the RIVER peer grief support model to other grievers who lost the same decedent upon request. We will offer study participation to the bereaved adult who the MEO obtains consent-to-contact, but remain flexible if that person feels another adult who is not the designated NOK would be a better fit for the study (e.g., someone who may benefit more from peer grief support). Participants will be people who (1) lost a loved one to an unintentional drug overdose death within the past year (2), are age 18 and older (3), fluently understand English or Spanish, and (4) have the capacity to give consent (e.g., excluding those with severe cognitive impairment, those in active psychosis, and those with developmental disabilities). Based on early feedback from our Medical Examiners Advisory Board, we will operationalize drug overdose deaths as those caused by intoxication from illicit substances (e.g., heroin), prescription substance misuse (e.g., using opioids in a way that was not prescribed), and/or counterfeit pills. Deaths in which intoxication exacerbated underlying medical conditions will be considered drug overdose deaths. Deaths that result from an adverse reaction to prescription medications that were used as prescribed (e.g., allergic reaction) will not be considered drug overdose deaths.

The study will exclude people who (1) need hospitalization for psychiatric symptoms or substance use disorders (2), have active suicidal ideation (3), lost someone to a drug overdose death more than one year ago, or (4) lost someone to a drug overdose death that was suicidal in nature or of undetermined intent. Those who use substances but do not require hospitalization for substance use disorder will *not* be excluded given the connection between substance use and poor grief outcomes. We will include only those grieving death that occurred within the past year because prolonged grief disorder can be diagnosed no earlier than 12-months after a loss [55] and we aim to evaluate the effectiveness of the RIVER peer grief support model in preventing this outcome. On the recommendation of our advisory boards, we will exclude people bereaved by suicide and deaths of undetermined intent because the bereavement experiences of these populations differ from those bereaved by unintentional drug overdose deaths.

**Fig. 2:**
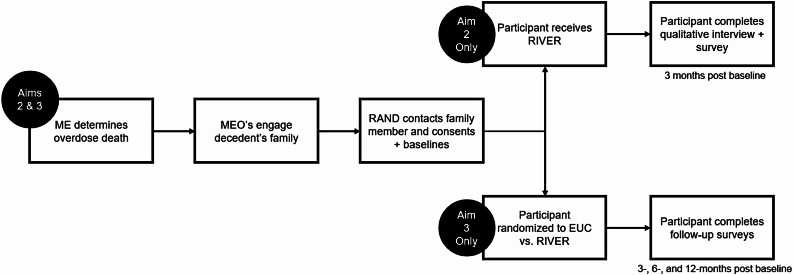
Pilot and RCT study design

We will proactively recruit people bereaved by overdose from MEOs. MEOs routinely identify and contact the legal next-of-kin to overdose decedents in order to provide them with death notifications. At the participating MEOs, nearly all manner and cause of death rulings are complete within 60 days of receiving the decedents’ remains. After this determination, staff at our partnering MEOs will ask families bereaved by overdose to give consent to be contacted by the study team. MEO or study staff will provide grievers with a postcard that describes the study, provides a QR code that links grievers with a page with additional study details and a brief animation video describing the study (see Fig. 2). Study engagement procedures will be carried out by MEO staff including medical examiners, death investigators, and chaplains.

Next, the study team will engage potentially eligible grievers. We will contact grievers to describe the study, screen them for eligibility, conduct the informed consent process, gather baseline data, and send the psychoeducational materials and list of local grief resources that make up EUC. The study team will contact families 30 to 90 days after they receive the death notification to contact grievers. This delay in outreach was recommended by PSCP and our advisory boards who suggested that grievers may be focused on making funeral arrangements and addressing immediate stabilization needs in the immediate aftermath of loss. However, grievers who contact the study team earlier may be enrolled earlier upon their request.

Enrolled participants will be referred to PSCP for peer grief support. PSCP will review grievers’ information and match them with a peer grief ally. Grievers are matched to an ally when possible, based on relationship to the descendent (e.g., parent vs. spouse) and geographical location (e.g., time zone). PSCP will contact the participant within three business days to schedule the first RIVER peer grief support session. During the first session, RIVER peer grief allies will schedule at least two additional sessions over the initial three months after enrollment at a cadence agreed-upon the peers and participants. All sessions will be conducted virtually or by phone. Sessions will be audio recorded to support peer grief ally supervision and fidelity assessment.

Three months after baseline, the study team will conduct 45-minute interviews with the participant. Participants will complete the Feasibility Intervention Measure (FIM) [56] that tests whether an intervention can be successfully used or carried out within a given setting. The FIM includes questions about how workable, possible, doable, and easy the RIVER peer grief support model was for the participant. Acceptability will be evaluated using the Acceptability of Intervention Measure [56] that tests how agreeable, palatable, or satisfactory the intervention was. Finally, we will use the Client Satisfaction Questionnaire (CSQ) [57, 58] where scores of at least 26 of 32 (81%) indicate acceptability of an intervention [59]. Questions include whether the intervention was a quality service, met their needs, and helped them cope. Participants will be asked open-ended questions about their experience with recruitment (e.g., “What did you like or not like about that process?”) and peer grief support (e.g., “What did you think of your discussions with your peer grief ally? How can we make the discussions helpful to people from different backgrounds?”). Interviews will be recorded and transcribed. Participants will be compensated $50 at baseline and $75 at the follow-up interview.

#### Aim 2 analyses

We will quantitatively analyze engagement, feasibility, acceptability, and satisfaction using summary statistics such as rates or means, as appropriate. Secondary analyses will explore differences in grief-related outcomes by number of peer grief sessions received by grievers; we will use linear regression to determine whether higher numbers of peer grief sessions are associated with better PGD and PGD-related outcomes. These analyses will help us to inform the minimum number of peer grief support sessions that will be used in this next phase.

Qualitative interviews will be coded by two coders to identify common themes using content analysis in Dedoose. We will also conduct a rapid content analysis [60] to efficiently glean insights that can be incorporated while still recruiting for the study. These methods include having a first coder writing detailed notes during each interview and categorizing across comments about the feasibility and acceptability of recruitment as well as the RIVER peer grief support model. Then, a second coder verifies the codes after listening to the interview recording. Analysts will meet regularly to discuss any differences and align on ratings. Coding will be iterative and will include comments that may be positive, negative, or neutral. Classic content analysis will be used to categorize quotes within each concept into themes (e.g., proactive outreach from the MEO was helpful) [61]. We chose a sample size of 20, however sample size will ultimately be determined by thematic saturation in qualitative analyses, at which point data collection becomes redundant [62]. If needed to form consensus themes, we will recruit additional participants for the pilot. We will follow procedures to maintain rigor of qualitative analyses, including an audit trail and data triangulation following the Standards for Reporting Qualitative Research (2014) [63] for data collection and analysis.

### Aim 3 procedures

We will recruit people who are bereaved by overdose from our three partnering MEOs for an RCT comparing the RIVER peer grief model plus EUC to EUC alone. Participants will complete assessments at baseline and 3-, 6-, and 12-month follow up. The inclusion and exclusion criteria for the RCT will be identical to Aim 2 unless advised otherwise by the advisory boards. We aim to recruit 320 people with a goal of 65% retention (208 people) at the 12-month follow-up.

We will also recruit the RIVER peer grief allies (up to 10) to complete a baseline and follow-up survey prior to and after the RCT. They will also be invited for a brief qualitative interview post-RCT to assess their experiences delivering peer grief support.

#### Randomization and stratification

Upon completion of the baseline survey, participants will be randomly assigned to the RIVER peer grief support model plus EUC or to EUC alone. Randomization will involve a stratified permuted block randomization with random size blocks. This ensures the number of people allocated to each group is approximately equal throughout recruitment [64]. We will stratify the sample by history of opioid misuse (lifetime heroin use and/or lifetime prescription opioid misuse) [65], under the premise that (1) drug overdose bereavement among PWUD may increase risk for future drug overdose deaths which typically involve opioids and (2) PWUD may experience grief problems that are more severe or different from people bereaved by a drug overdose death who do not use drugs. Randomization will be performed within each stratum.

#### Measures

**Fig. 3:**
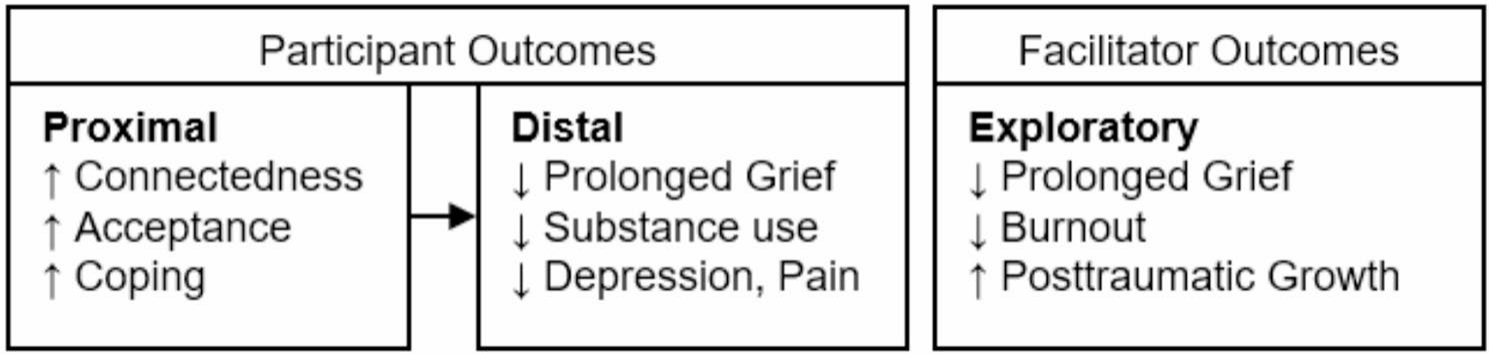
Hypothesized outcomes

All measures will be collected using surveys administered by an interviewer over the phone. We have selected measures that are reliable and valid in populations experiencing grief, as well as measures from the NIH HEAL Initiative Common Data Elements Repository and the NIH PhenX Toolkit [66] (see Table 1). Our primary outcome is PGD, and the associated outcomes that we are evaluating have previously demonstrated a relationship with the severity of grief symptoms experienced. We also have additional proximal and distal outcomes that will serve as pre-specified secondary outcomes (see Table 1; Fig. 3). Other potential covariates include participants’ relationship to the decedent, time since the death, history of exposure to other drug overdose deaths (i.e., number of people they knew who died by overdose), drug of choice (for PWUD enrolled in the RCT), and service utilization (measured using the NIH PhenX Access to Health Services Protocol derived from the National Health Interview Survey [67]).

**Table 1:** Outcomes

**RIVER Feasibility, Acceptability, and Satisfaction (Aim 2)**		
Feasibility	Extent of how workable, possible, doable, and easy RIVER was	Feasibility Intervention Measure (FIM)
Acceptability	Extent of how agreeable, palatable, or satisfactory RIVER was	Acceptability of Intervention Measure
Satisfaction	Assesses RIVER on quality, needs met, and helpfulness	Client Satisfaction Questionnaire
**Distal & Proximal Outcomes (Aim 3)**		
Grief Symptoms (Primary)	Presence and extent of persistent emotional, cognitive, and behavioral responses to the loss by the griever (e.g., intense yearning, preoccupation with the deceased, difficulty accepting the death, and disruptions in daily functioning)	Brief Grief Questionnaire (BGQ)
Connectedness	Extent to which individual still feels bonded to their deceased loved one and the people in their community	Continuing Bonds Scale Multidimensional Scale of Perceived Social Support\
Grief Acceptance	Presence and extent of adaptive beliefs that support the griever in acknowledging the loss, tolerating painful emotions, maintaining a meaningful connection with the deceased, and re-engaging in their daily life	Typical Beliefs Questionnaire
Coping Strategies	Cognitive, behavioral, social, and spiritual tools the griever uses to manage the emotional, practical, and existential challenges that arise after a loss	The Coping Assessment for Bereavement and Loss Experiences (CABLE)
Grief-related Avoidance	Presence and extent of efforts used by the griever to evade external and internal reminders of the loss (e.g., certain places, activities, thoughts, or emotions about the deceased) and reduce distress	Grief-Related Avoidance Questionnaire (GRAQ)
**Secondary Outcomes (Aim 3)**		
Substance Use	Presence and frequency of substance use, and substance use disorders in the past 30 days	Tobacco, Alcohol, Prescription Medication, and Other Substances Tool (TAPS) PROMIS
Depression	Presence and severity of symptoms related to Major Depressive Disorder (e.g., persistent sadness, anhedonia, changes in sleep and appetite, and suicidal ideation)	Patient Health Quesitonnaire-9 (PHQ-9)
Posttraumatic Stress Disorder	Presence of certain experiences related to the loss by the griever (e.g., nightmares about the loss, avoidance of reminders of the loss, hypervigilance, emotional or social detachment, and guilt or blame about the loss) over the past month that are associated with posttraumatic stress disorder	Primary Care PTSD Screen for DSM-5 (PC-PTSD-5)
Physical Pain	The intensity of physical pain experienced by the griever and the level of interference on their daily life and wellbeing	Pain, Enjoyment of Life and General Activity Scale (PEG)
**RIVER Peer Grief Ally Outcomes (Aim 3)**		
Grief Symptoms	Presence and extent of persistent emotional, cognitive, and behavioral responses to the loss by the griever (e.g., intense yearning, preoccupation with the deceased, difficulty accepting the death, and disruptions in daily functioning)	Brief Grief Questionnaire (BGQ; same measure used in the primary outcome with grievers)
Posttraumatic Growth	Presence and extent of positive psychological changes following overdose bereavement that reflect a greater appreciation of life, improved relationships, increased personal strength, and an openness to new possibilities	Posttraumatic Growth Inventory (PTGI)
Burnout	Presence and extent of physical and emotional exhaustion as a result of prolonged stress and work demands that is characterized by fatigue, reduced energy, and diminished motivation	Copenhagen Burnout Inventory

### Data management and analysis plan

Aim 3 will test the effect of receiving the RIVER peer grief support model plus EUC compared to EUC alone by contrasting outcomes of grief and using regression-based modeling to leverage all time points while adjusting for the stratification variable. We will use Intent-to-Treat analyses. Hypotheses regarding the effectiveness of the RIVER peer grief support approach will be tested with a repeated measures mixed-effects linear model, with experimental group, time (4 assessment time points), and their interaction as fixed effects. Inclusion of the interaction term will allow for examination of the difference in response trajectories between intervention arms. We will also include the stratification variable as a covariate in the model. For missing data, we will proceed with likelihood-based mixed-effects models, which are valid under the Missing at Random (MAR) assumption. For non-ignorable missingness, we will add sensitivity analyses. Qualitative interview data with peer grief allies will also be used to contextualize quantitative results. For example, if allies share that support was more or less challenging for certain groups, we may explore potential moderators of the treatment effect.

### Power

We powered our trial to detect a moderate effect size based on arm-differences of proportions of prolonged grief, due to the dearth of pilot data on mean differences. We considered an 18% arm difference in rates of prolonged grief symptoms, assuming 27% in the RIVER plus EUC arm and 45% in the EUC only arm. Since the RIVER peer grief support model has not yet been evaluated in those specifically bereaved by drug overdose deaths, we conservatively used estimates of proportions reported on rates of PGD among those bereaved by suicide [6]. We will have 80% power to detect a moderate effect size of a Cohen’s d of 0.4. We based this calculation on a two-tailed test of proportions for the null hypothesis of no difference in arms, and a significance level of 0.05. We are also adequately powered to perform repeated measures analysis to detect a group by time interaction term in a mixed model to address Aim 3. With our proposed follow-up sample size (*n* = 208), we have 90% power to detect a group by time interaction, expecting a Cohen’s d of 0.4 as before, and assuming adjustment of the baseline grief score, and a correlation of 50% between repeated measures.

### Discussion

Our nation is experiencing historic rates of drug overdose deaths that have left behind millions of bereaved families. People who are bereaved by overdose experience a “special grief” that compounds suffering and help-seeking. Overdose bereavement is an unfortunate and common reality for many Americans, and the ripple effect of overdose deaths may increase risk for overdose morbidity and mortality among the bereaved. No evidence-based interventions exist to support this population and the science must catch up. Peer grief support interventions hold promise and may be especially crucial for this population given the increased stigma, isolation, and risks this population faces. This study seeks not only to understand whether a peer support model aids those grieving an overdose death, but also to explore the feasibility and acceptability of proactively engaging grievers through MEOs. In doing so, we aim to reach out to grievers who may not otherwise seek care. If our study is successful, our practice-research partnership and engagement of advisory board members will propel the translation of research to practice and address the large toll the overdose crisis takes on this overlooked population.

## Data Availability

No datasets were generated or analysed during the current study.
